# Screening of Tumor Suppressor Genes in Metastatic Colorectal Cancer

**DOI:** 10.1155/2017/2769140

**Published:** 2017-04-04

**Authors:** Lu Qi, Yanqing Ding

**Affiliations:** ^1^Department of Pathology, School of Basic Medical Sciences, Southern Medical University, Guangzhou 510515, China; ^2^Department of Pathology, Nanfang Hospital, Southern Medical University, Guangzhou 510515, China

## Abstract

Most tumor suppressor genes are commonly inactivated in the development of colorectal cancer (CRC). The activation of tumor suppressor genes may be beneficial to suppress the development and metastasis of CRC. This study analyzed genes expression and methylation levels in different stages of CRC. Genes with downregulated mRNA expression and upregulated methylation level in advanced CRC were screened as the potential tumor suppressor genes. After comparing the methylation level of screened genes, we found that* MBD1* gene had downregulated mRNA expression and upregulated methylation levels in advanced CRC and continuously upregulated methylation level in the progression of CRC. Enrichment analysis revealed that genes expression in accordance with the elevated expression of* MBD1* mainly located on chromosomes 17p13 and 17p12 and 8 tumor suppressor genes located on chromosome 17p13. Further enrichment analysis of transcription factor binding site identified that SP1 binding site had higher enrichment and could bind with* MBD1*. In conclusion,* MBD1* may be a tumor suppressor gene in advanced CRC and affect the development and metastasis of CRC by regulating 8 tumor suppressor genes through binding with SP1.

## 1. Introduction

The occurrence and development of malignant tumors are mainly caused by the activation of oncogene and inactivation of tumor suppressor genes. In colorectal cancer (CRC), tumor suppressor genes are significantly inhibited with the progression of cancer. Screening genes with the function of inhibiting tumor growth in advanced CRC is helpful to find the way of suppressing the progression and metastasis of tumor. A gene is a locus (or region) of DNA which is made up of nucleotides and produces biological function through gene expression and protein coding. In the progression of cancer, the expression of oncogenes is enhanced, while the expression of tumor suppressor gene is inhibited. Methylation modification is essential for gene expression. DNA methylation can shut down the activity of certain genes, while demethylation promotes the activity of genes. Hence, the change of methylation levels is a critical index in the process of screening tumor suppressor genes. In advanced CRC, tumor cell can metastasize to lymph nodes and even distant organ. In this stage, the activity of tumor suppressor gene is significantly inhibited. Therefore, the activation of tumor suppressor genes may be the potential way for the treatment of CRC. This study analyzed genes expression and methylation levels in the different stage of CRC to find out genes with decreased expressions and increased methylation levels in advanced CRC. By comparing methylation levels in different stage, gene with continuously upregulation methylation levels in the progression of CRC was screened. After screening,* MBD1* may be a tumor suppressor gene in advanced CRC. In order to figure out the potential mechanism of* MBD1* in the process of tumor inhibition, this study further screened genes related to* MBD1 *high expression in CRC gene expression profile.

## 2. Methods and Materials

### 2.1. Data Source

DNA methylation data of CRC was from Level 3 Cancer Genome Altas (TCGA) [1], with Illumina Human Methylation 27 as the data chip platform, including 37 cases of normal CRC data and 166 cases of CRC data. The 166 cases of CRC data included 32 cases of stage I data, 64 of stage II data, 45 of stage III data, 24 of stage IV data, and 1 case with unclear stage. This study mainly analyzed the methylation data of the 165 CRC cases.

CRC gene expression profile data were taken from TCGA, with Agilent G4502A as the data chip platform, including 174 cases of expression profile data (19 cases of normal CRC and 155 cases of CRC) (Table 1).

### 2.2. Gene Screening with Differential Methylation and Expression

According to AJCC stage, early CRC data (stage I and stage II) and metastatic CRC data (stage III and stage IV) were compared with 37 cases of normal CRC data to screen genes with differential methylation level. In the analysis of gene expression, based on the 155 cases of CRC expression profile data, early CRC data (stage I data and stage II) and metastatic CRC data (stage III and stage IV) were compared with 19 cases of normal CRC data to screen genes with differential expression. Unpaired *t*-test was used for gene screening. Bonferroni FWER [2] was performed for adjusting *p* value to control false discovery rate (FDR). *p* value less than 0.05 was considered significant difference.

CRC methylation data were divided into four groups (I, II, III, and IV stage) in order to screen genes with continuous increase or decrease of methylation level at the four stages. Multiple comparisons were performed for differential gene expression screening followed by paired comparison (stage II versus stage I; stage III versus stage II; stage IV versus stage III) by using One-way ANOVA. The folder change of differential gene was 1 time. Bonferroni FWER was performed to adjust *p* value. *p* value less than 0.05 was considered significant difference. The intersection was taken from the three paired differential genes. Based on the progression of CRC, genes with continuous increase or decrease of methylation level at the four stages were selected.

Bonferroni FWER was a step-wise process, in an individual correction way for each *p* value and multiplying each gene's *p* value by its number on gene lists. It shows significance if the corrected *p* value was less than the error ratio. The corrected *p* value equals the multiplication of *p* value by gene number and is less than 0.05. Therefore, if 1000 genes were tested each time, single *p* value accepted was 0.00005 at most, thus making correction strict. We did so by using Bonferroni FWER to correct *p* value to ensure reliability of the screened out difference genes, especially when colorectal cancer tissues had less difference.

### 2.3. Regulation Mechanism of* MBD1* in CRC

Expression profile data GSE39582 (including 566 cases of CRC tissues) taken from Gene Expression Omnibus (GEO) [3] were used to analyze the potential mechanism of* MBD1* in CRC. To clarify the effect of high and low expression of* MBD1* on CRC expression profile, 566 cases of expression profile data were divided into high and low groups according to the expression of* MBD1* in CRC: expression of* MBD1* was ordered from high to low and divided into two groups (*MBD1* high expression* and MBD1* low expression) with 283 cases in each group; GSEA [4, 5] was used for enrichment analysis between the two group based on chromosome location (gene set, version V5.1).

## 3. Results

### 3.1. Screening Results of Tumor Suppressor Gene

To screen genes with the function of tumor inhibition in advanced CRC, only genes with downregulated mRNA expression and upregulated methylation level in metastatic CRC were selected. Based on the data from TCGA database, early CRC data (stage I data and stage II) and metastatic CRC data (stage III and stage IV) were compared with normal CRC data and finally 1470 genes with upregulated methylation level, 2677 genes with downregulated methylation level, 1984 genes with upregulated expression, and 3255 genes with downregulated expression were obtained in early CRC. There were 1164 genes with upregulated methylation level, 2569 genes with downregulated methylation level, 1882 genes with upregulated expression, and 3143 genes with downregulated expression obtained in advanced CRC.

The intersection analysis was performed based on the differential expression genes and differential methylation genes and divided into four groups: (1) intersection was obtained from genes with upregulated methylation level in early stage and in advanced stage; 355 specific genes with upregulated methylation in early stage; and 49 specific genes with upregulated methylation in advanced stage and 1115 common genes were collected; (2) intersection was obtained from genes with downregulated methylation level in early stage and in advanced stage; 522 specific genes with downregulated methylation in early stage; and 414 specific genes with downregulated methylation in advanced stage and 2155 common genes were collected; (3) intersection was obtained from genes with upregulated gene expression in early stage and in advanced stage; 315 specific genes with upregulated expression in early stage, and 213 specific genes with upregulated expression in advanced stage and 1669 common genes were collected; (4) intersection was obtained from genes with downregulated gene expression in early stage and in advanced stage; 428 specific genes with downregulated expression in early stage, and 316 specific genes with downregulated expression in advanced stage and 2827 common genes were collected.

Intersection was performed in genes with upregulated methylation and downregulated gene expression in early stage, and 15 genes were obtained. Intersection was performed in genes with downregulated methylation and upregulated expression in early stage, and 7 genes were obtained. Intersection was performed in genes with downregulated methylation and upregulated expression in advanced stage, and 8 genes were obtained. Intersection was performed in genes with upregulated methylation and downregulated expression in advanced stage, and 3 genes were obtained.

Upregulation of gene expression can promote the performance of gene function, while upregulation of methylation can inhibit gene function. Hence, only the 15 genes with upregulated methylation and downregulated expression in early stage might be the tumor suppressor genes. The 7 genes with downregulated methylation and upregulated expression in early stage might be the cancer-promoting genes. The 8 genes with downregulated methylation and upregulated expression in advanced stage might be the cancer-promoting genes. The 3 genes with upregulated methylation and downregulated expression in advanced stage might be the tumor suppressor genes. From the above results, we found that tumor suppressor gene was more while cancer-promoting gene was less in early stage. In advanced stage, cancer-promoting gene was more while tumor suppressor gene was less.

**Figure 1:**
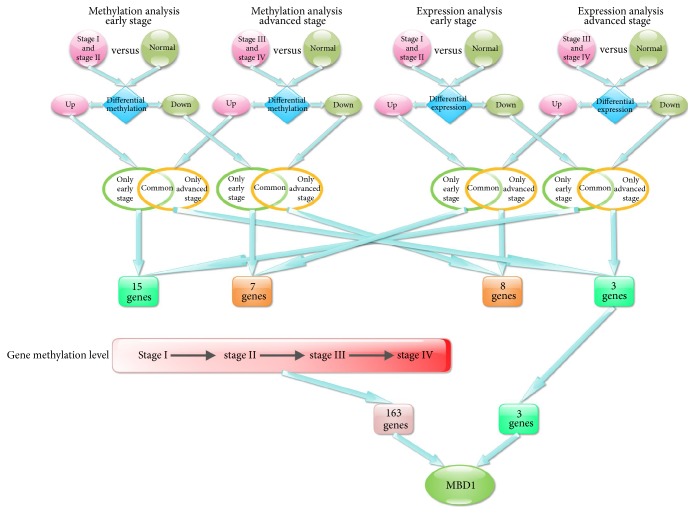
Flow chart of screening differentially methylated genes and differentially expressed genes.

From the above analysis, we only found 3 genes with upregulated methylation and downregulated gene expression in advanced CRC. In order to screen genes with sustained change of methylation in the four stages of CRC, we compared genes with differential methylation in three paired groups: stage II versus stage I, stage III versus stage II and stage IV versus stage III and obtained the intersection. A total of 163 genes with sustained increasing methylation and 222 genes with sustained decreasing methylation were screened in I, II, III, and IV stages of CRC. Intersection was obtained among the 163 genes with increased methylation in CRC and 3 genes with upregulated methylation and downregulated gene expression in advanced CRC, and finally* MBD1* was screened (Figure 1).

### 3.2. Survival Analysis of* MBD1* in CRC

**Figure 2:**
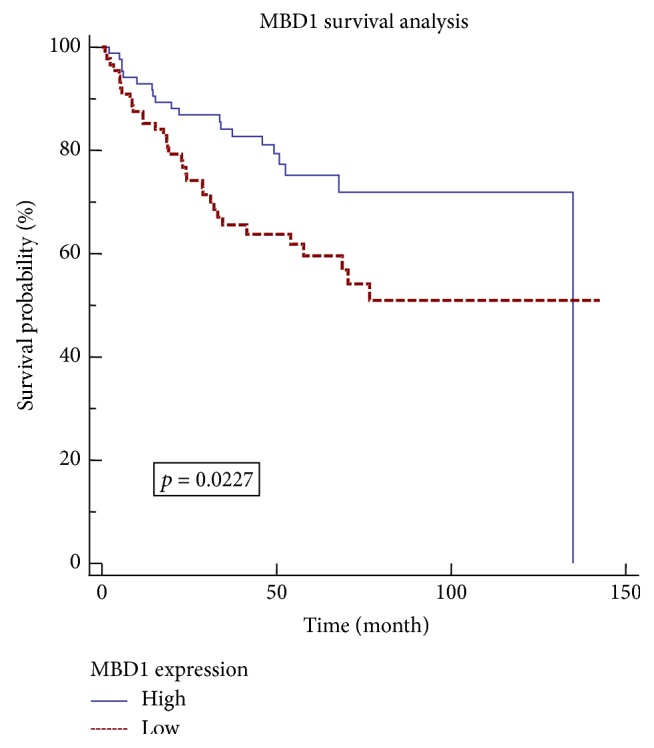
Survival analysis diagram of MBD1.

*MBD1* had upregulated methylation level and downregulated gene expression in advanced CRC and had sustained increasing methylation in the four stages of CRC. Hence,* MBD1* might be a tumor suppressor gene in advanced CRC. For further confirmation, expression profile data GSE17536 taken from GEO database was used for survival analysis of* MBD1*. GSE17536 data includes 177 CRC tissues with determined survival time, with Affymetrix Human Genome U133 Plus 2.0 Array as the chip platform [6]. We extracted 177 expression values of* MBD1*, ordered and divided into* MBD1* high-expression group and* MBD1* low-expression group. Each group included 88 cases of data, with the median data (1 case) being deleted. Kaplan-Meier [7] was used to analyze the survival rate of the two groups with the survival curve drawn (Figure 2). The results showed that, with the change of* MBD1* expression, the difference of survival rate was significant (*p* = 0.0227). Patients with high-expression* MBD1* had higher survival rate than patients with low-expression* MBD1*.

### 3.3. Regulation Mechanism of* MBD1* in CRC

**Figure 3:**
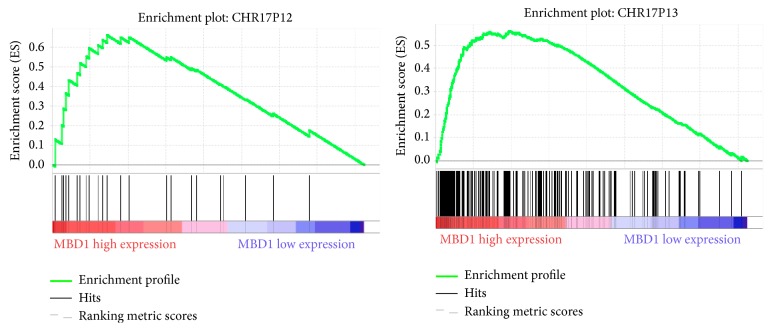
Enrichment figure of gene chromosomes 17 p12 and 17 p13 region that related to MBD1 expression.

**Figure 4:**
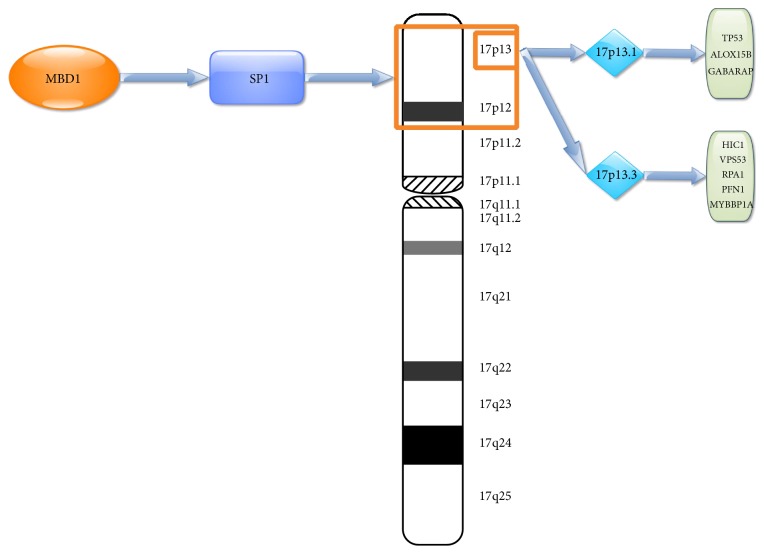
Regulating schema diagram of 8 tumor suppressor genes that are regulated by MBD1 through binding with SP1.

In order to further confirm the molecular mechanism of* MBD1* in CRC, expression profile data from 566 CRC cases at different stages were divided in the* MBD1* high-expression group and* MBD1* low-expression group and GSEA enrichment analysis was performed based on chromosome location. The results revealed that two gene sets with high enrichment in the* MBD1* high-expression group were located in chromosomes 17P13 (*p* = 0.002) and 17P12 (*p* = 0.002) (Figure 3). The expressions of the two gene set were upregulated in the* MBD1* high-expression group, suggesting that 17P13 and 17P12 might be the chromosome zone where* MBD1* worked. A total of 89 genes contributed to the enrichment of 17P13 gene set and 12 genes contributed to the enrichment of 17P12 gene set. A total of 1217 known tumor suppressor genes was searched from the tumor suppressor gene database (TSGene 2.0) [8, 9]. Intersection was performed among the 1217 genes and previously mentioned 89 genes, and 8 tumor suppressor genes (*HIC1, VPS53, RPA1, ALOX15B, TP53, PFN1, MYBBP1A*, and* GABARAP*) were obtained. No intersection was found among the 1217 genes and previously mentioned 12 genes. Because of all the 8 genes located 17P13 zone, a total of 89 genes in the region of 17P13 were performed through CYTOBAND database of DAVID [10, 11] and 17P13.3 and 17P13.1 had higher enrichment. 17P13.3 enrichment included 22 genes, including 5 tumor suppressor genes (*HIC1, VPS53, RPA1, PFN1*, and* MYBBP1A*). 17P13.1 enrichment included 27 genes, including 3 tumor suppressor genes (*ALOX15B, TP53*, and* GABARAP*). In order to clarify the potential mechanism of the 8 tumor suppressor genes, enrichment analysis of transcription factor binding sites was performed by using TFM-Explorer [12] in 22 genes in 17P13.3 zone and 27 genes in 17P13.1 zone. A total of 29 proteins interacted with* MBD1* after being verified by BioGRID [13] database search. Intersection was obtained based on the 29 genes and the enrichment of transcription factors, SP1, was screened. The binding site of SP1 had higher enrichment in the upstream regulation region of 17P13.3 and 17P13.1 and the upstream regulation region of the 8 tumor suppressor genes all found SP1 binding site. Moreover, SP1 could bind with* MBD1*. Hence,* MBD1* may be a tumor suppressor gene in advanced CRC and affect the development and metastasis of CRC by regulating 8 tumor suppressor genes through binding with SP1 (Figure 4).

## 4. Discussion

MBD1, named methyl-CpG binding protein 1, is a transcriptional repressor and belongs to one member of methyl-CpG binding domain (MBD) family. MBD1 can negatively regulate the transcriptional activity of RNA polymerase II promoter and be involved in chromosome silencing dependent by methylation. Hence, MBD1 can regulate both gene expression and methylation level. Previous study reported that the upregulation of* MBD1* enhanced the epithelial mesenchymal transition and invasion of pancreatic cancer cells [14], which suggested that* MBD1* contributed to the tumor growth in pancreatic cancer. Another study reported that deletion of 18q21 chromosome where* MBD1* was located occurred frequently in CRC [15, 16], which suggested that genes in 18q21 chromosome zone might inhibit tumor growth in CRC. The deletion of 18q21 chromosome could result in the abnormal activation of cancer genes.* MBD1* might be a tumor suppressor gene for its location. Previous studies demonstrated that the polymorphism of* MBD1* was related to the risk of lung cancer [17, 18], which provided evidence that* MBD1* might inhibit tumor growth in lung cancer. Hence,* MBD1* expression may have different biological effect in different cancer. In this study,* MBD1* had upregulated methylation and downregulated gene expression in advanced CRC and also had sustained upregulated methylation in the four stages of CRC, which suggested that* MBD1* was a tumor suppressor gene for advanced CRC. In the study of expression profile of CRC, we found that high expression of* MBD1* was closely related to genes in chromosomes 17P12 and 17P13 and the two chromosome zones included many known tumor suppressor genes, such as* HIC1, VPS53, RPA1, ALOX15B, TP53, PFN1, MYBBP1A*, and* GABARAP*. Many studies have been reported that* TP53* was a tumor suppressor gene. Further study found that the upstream regulation region of the 8 tumor suppressor genes all found SP1 binding site. Moreover, SP1 could bind with* MBD1*. Hence,* MBD1* may be a tumor suppressor gene in advanced CRC and affect the development and metastasis of CRC by regulating 8 tumor suppressor genes through binding with SP1. This study determined that* MBD1* may be a tumor suppressor gene in advanced CRC from the aspect of gene expression and methylation level and speculated that* MBD1* could regulate chromosomes 17P12 and 17P13 zones to further regulate the expressions of tumor suppressor genes. Therefore, the activation of* MBD1* in advanced CRC may inhibit and even reverse the development and metastasis of CRC.

## Figures and Tables

**Table 1:** The distribution of clinical characteristics of methylation and expression profile in CRC patients.

Characteristics	Colorectal cancer	
	Methylation	Expression
Sum	166	155
Gender		
Male	83	79
Female	83	76
Age at initial pathologic diagnosis		
<50	12	10
≥50	154	145
Anatomic neoplasm subdivision		
Ascending colon	32	28
Cecum	33	29
Descending colon	6	6
Hepatic flexure	8	9
Sigmoid colon	69	66
Splenic flexure	2	2
Transverse colon	15	14
TNM		
T1 + T2 + T3 + T4	166	155
N1 + N2	67	60
M1	24	23
AJCC pathologic tumor_stage		
Stage I	32	29
Stage II	64	63
Stage III	45	39
Stage IV	24	23
Histologic diagnosis		
Colon adenocarcinoma	140	131
Colon mucinous adenocarcinoma	24	22
Family history colorectal cancer		
Yes	26	23
No	140	132
History of another malignancy		
Yes	9	9
No	157	146
History colon polyps		
Yes	85	84
No	81	71
Tumor status		
Tumor free	10	10
With tumor	155	144
Vascular invasion indicator		
Yes	37	34
No	106	107
Lymphovascular invasion indicator		
Yes	87	78
No	70	72
